# Co-infection and enterovirus B: post EV-A71 mass vaccination scenario in China

**DOI:** 10.1186/s12879-022-07661-3

**Published:** 2022-08-04

**Authors:** Wei Guo, Danhan Xu, Shanri Cong, Zengqing Du, Li Li, Ming Zhang, Changzeng Feng, Guohong Bao, Hao Sun, Zhaoqing Yang, Shaohui Ma

**Affiliations:** 1 Institute of Medical Biology, Chinese Academy of Medical Sciences, and Peking Union Medical College (CAMS & PUMC), 935 Jiao Ling Road, Kunming, 650118 Yunnan Province People’s Republic of China; 2 Yunnan Key Laboratory of Vaccine Research Development on Severe Infectious Disease, Kunming, 650118 People’s Republic of China; 3 Department of Infectious Diseases, Kunming Children’s Hospital, Kunming, China; 4 Department of Clinical Laboratory, Kunming Maternal and Child Health Hospital, Kunming, 650031 China; 5 First People’s Hospital of Yunnan Province, Kunming, 650032 People’s Republic of China

**Keywords:** Hand, foot, and mouth disease, Co-circulation, Co-infection, Enterovirus

## Abstract

**Background:**

Hand, foot, and mouth disease (HFMD) is a common child infectious disease caused by more than 20 enterovirus (EV) serotypes. In recent years, *enterovirus A71* (EV-A71) has been replaced by *Coxsackievirus A6* (CV-A6) to become the predominant serotype. Multiple EV serotypes co-circulate in HFMD epidemics, and this study aimed to investigate the etiological epidemic characteristics of an HFMD outbreak in Kunming, China in 2019.

**Methods:**

The clinical samples of 459 EV-associated HFMD patients in 2019 were used to amplify the VP1 gene region by the three sets of primers and identify serotypes using the molecular biology method. Phylogenetic analyses were performed based on the VP1 gene.

**Results:**

Three hundred and forty-eight cases out of 459 HFMD patients were confirmed as EV infection. Of these 191 (41.61%) were single EV infections and 34.20% had co-infections. The EVs were assigned to 18 EV serotypes, of which CV-A6 was predominant (11.33%), followed by CV-B1 (8.93%), CV-A4 (5.23%), CV-A9 (4.58%), CV-A 16 (3.49%) and CV-A10 and CVA5 both 1.96%. Co-infection of CV-A6 with other EVs was present in 15.25% of these cases, followed by co-infection with CV-A16 and other EVs. The VP1 sequences used in the phylogenetic analyses showed that the CV-A6, CV-B1 and CV-A4 sequences belonged to the sub-genogroup D3 and genogroups F and E, respectively.

**Conclusion:**

Co-circulation and co-infection of multiple serotypes were the etiological characteristic of the HFMD epidemic in Kunming China in 2019 with CV-A-6, CV-B1 and CV-A4 as the predominant serotypes. This is the first report of CV-B1 as a predominant serotype in China and may provide valuable information for the diagnosis, prevention and control of HFMD.

## Background

The genus *Enterovirus* (EVs) are categorized into fifteen species including EV-A, B, C, D, E, F, G, H, I, J, K and L and Rhinovirus A, B and C within the family *Picornaviridae*, including 274 serotypes (www.picornaviridae.com/sg3_ensavirinae/enterovirus/enterovirus.htm). Since 2008, hand, foot and mouth disease (HFMD) has been a common children’s disease in China, caused by EV-A, EV-B, and EV-C [1]. The disease is characterized by fever and vesicular eruptions on the hands, feet and oral mucosa, but some patients can develop severe complications including encephalitis, meningitis, brainstem encephalitis, acute flaccid paralysis, cardiorespiratory failure and death [2, 3]. Although China has had access to an EV-A71 vaccine since 2016, the incidence of HFMD is still extremely high [4]. Currently *Coxsackieviruses* (CV)-A6 and -A10 have become the predominant serotypes, causing mild, severe and fatal cases of HFMD [4]. Co-circulation of multi-serotypes has become an epidemiological feature of FHMD outbreaks [4, 5], but the serotypes have no cross protection. To fully control HFMD caused by multiple serotypes, a multivalent vaccine needs to be developed. Epidemic and pathogen surveillance can provide valuable information on measures to prevent and control HFMD and on the formulation of multivalent vaccines.

The purpose of this study was to investigate the etiology spectrum after mass vaccination using an EV-A71 vaccine, combining three sets of primers to analyze the etiological characteristics of EVs in HFMD specimens. By analyzing the prevalence of pathogens, changes in the etiological spectrum can be monitored and strategies provided for developing multivalent vaccines to prevent HFMD.

## Methods

### Patients and clinical sample collection

Kunming City is a tourist city in the southwest region of China, covering an area of 21,473 km [2] and has a population of 6,950,000. The annual average temperature is 15 °C. A total of 459 stool specimens from children admitted with HFMD were collected in 2019, when the data of the patient’s personal details and clinical symptoms was collected, and the stool specimens obtained were stored at − 80 °C. The clinical and laboratory diagnoses of cases were based on the diagnostic criteria in the Guidelines for the Diagnosis and Treatment of HFMD 2018 edition (China) [6].

### Nucleotide acid detection by real time polymerase chain reaction (RT–PCR)

Viral RNAs were extracted using with a QIAamp Viral RNA Mini Kit (Qiagen, Valencia, CA, USA). The detection of pan-enteroviruses, EV-A71, CV-A16, CV-A6, and CV-A10 was performed in the Applied Biosystems-7500 system by one-step RT–PCR assay (Daan Biotech, Guangzhou, China). The RT-PCR was conducted based on the standard protocol as 50 °C for 30 min, 95 °C for 5 min and then followed by 45 cycles with 95 °C at 10 s and 55 °C at 40 s. Samples with a CT (Cycle Threshold) value less than 43 were considered positive.

### Amplification of the partial VP1 regions and sequencing

To further identify the serotypes according to the standard protocol, three half-nested and nested primers are used to amplify the VP1 gene region of a specimen [7–9]. One gram of stool specimen was suspended in 5 mL of phosphate buffered saline (PBS) and the suspension was centrifuged at 4 °C, 2000×*g* for 30 min. According to the manufacturer’s recommended procedure, a QIAamp Viral RNA Mini Kit (Qiagen, Valencia, CA, USA) was used to extract viral RNA. Reverse transcription polymerase chain reaction (RT-PCR) and PCR were conducted using a PrimeScript One-Step RT-PCR kit ver.2 (Takara, China) and Pyrobest™ DNA Polymerase (TaKaRa, China). The first round of RT-PCR was performed in a 25 µL reaction containing 8 µL RNA, 1 µL RT-PCR mix (Takara, China), 12.5 µL of 2× reaction buffer, and 20 pmol each of the outer primers for EV-A or EV-B or 222 and 224. The amplification program was 50 °C for 30 min, 94 °C for 2 min, followed by 30 cycles of 94 °C for 30 s, 52 °C for 30 s and 72 °C for 1 min. The second round of PCR was performed with 5 µL of the first-round PCR products and 20 pmol of each of the inner primers in a volume of 25 µL under the same PCR conditions described above. The positive PCR products were purified and bidirectionally sequenced on an ABI3730XL automatic sequencer (Applied Biosystems, Foster City, CA, USA). The obtained sequences were used for molecular typing with the online Enterovirus Genotyping Tool version 0.1.

### Sequence analysis and phylogenetic analysis

Multiple partial VP1 sequences were aligned at BLAST (https://blast.ncbi.nlm.nih.gov/Blast.cgi). Geneious 5.4.1 software was used for nucleotide sequence alignment and homology comparison. The phylogenetic analysis based on the VP1 sequence was conducted in the MEGA 6 program, using the neighbor-joining method (NJ) and the maximum comprehensive likelihood method as well as the Kimura two-parameter model [6]. A Bootstrap value exceeding 75% was considered statistically significant. A nucleotide difference of more than 15% between groups in the VP1 region was used to distinguish genotypes.

### Statistical analysis

The data were analyzed using the SPSS 26.0 statistical software (SSPS, Inc, Chicago, IL). Data were analyzed by a one-way analysis of variance test. *P* < 0.05 were considered statistically significant.

### Nucleotide sequence accession number

The VP1 sequences of the EV strains obtained in this study were deposited in the GenBank database under the accession numbers: OK315571-OK315637.

## Results

### Cases and epidemiology

A total of 459 cases of HFMD were reported during the study period and 348 (75.82%) were positive for EVs. The sex ratio was 1.40:1, with 268 patients as boys and 191 were girls. The average age of the patients (± SD) is 2.8 (± 1.90) years. And all patients in the study previously were not vaccinated with the EV-A71 vaccine.

### Pathogenic characteristics

Cases of infection due to EV were 348 (75.82%) of which 191 (41.61%) were a single EV infection and detected EVs belonged to 18 EV serotypes. Of these, CV-A6 was predominant, accounting for 11.33% of the cases, followed by CV-B1 (8.93%), CV-A4 (5.23%), CV-A9 (4.58%), CV-A16 (3.49%) and CV-A10 and CV-A5, both 1.96%.

Co-infection accounted for 34.20% of cases. Except for EV-A71 and CV-A16, the co-infection of CV-A6 and other EVs dominates (15.25%), followed by the co-infection of CV-A16 and other EVs (10.02%, except for EV-A71 and CV-A6), and co-infection of other EVs (5.01%). In severe cases, co-infection of CV-A16 and other EVs dominates, followed by co-infection of CV-A6 and other EVs, except for EV-A71 and CV-A16) co-infection of EV-A71 and other EVs, except Co-infection of CV-A6 and CV-A16 and other EVs (Table 1).

**Table 1:** The pathogens identified in patients with hand-foot-mouth disease in Yunnan, China, 2019

Virus	Total (n = 459)		*P*
	No	%	
Enterovirus infection	348	75.82	
Average age	2.80 ± 1.90		
Single enterovirus infection	191	41.61	< 0.01
Enterovirus A	119	25.9	
CVA6	52	11.33	< 0.01
CVA4	24	5.23	< 0.01
CVA16	16	3.49	< 0.01
CVA10	9	1.96	0.786
CVA5	9	1.96	–
EV71	2	0.44	< 0.01
Other Enterovirus A	9	1.96	0.786
Enterovirus B	72	15.7	
CVB1	41	8.93	< 0.01
CVA9	21	4.58	< 0.01
Other Enterovirus B	10	2.18	0.031
Co-infection	157	34.20	< 0.01
EV71 + other EVs (except CVA6 + CVA16)	9	1.96	**–**
CVA6 + other EVs (except EV71 + CVA16)	70	15.25	< 0.01
CVA6 + CVA16 + other EVs (except EV71)	7	1.53	0.661
CVA16 + other EVs (except EV71 + CVA6)	46	10.02	< 0.01
Other EVs	25	5.01	< 0.01
Non-enterovirus infection	111	24.18	

Other Enterovirus A: CVA8 (n = 3), CVA2 (n = 2) and CVA12 (n = 2) ; Other Enterovirus B: E18 (n = 4), CVB2 (n = 1), CVB3 (n = 1), CVB4 (n = 1), E1 (n = 1), E6 (n = 1) and E30 (n = 1); –: Using the values of CVA5 and EV71 + other EVs (except CVA6 + CVA16) as references

### Phylogenetic analysis

**Fig. 1:**
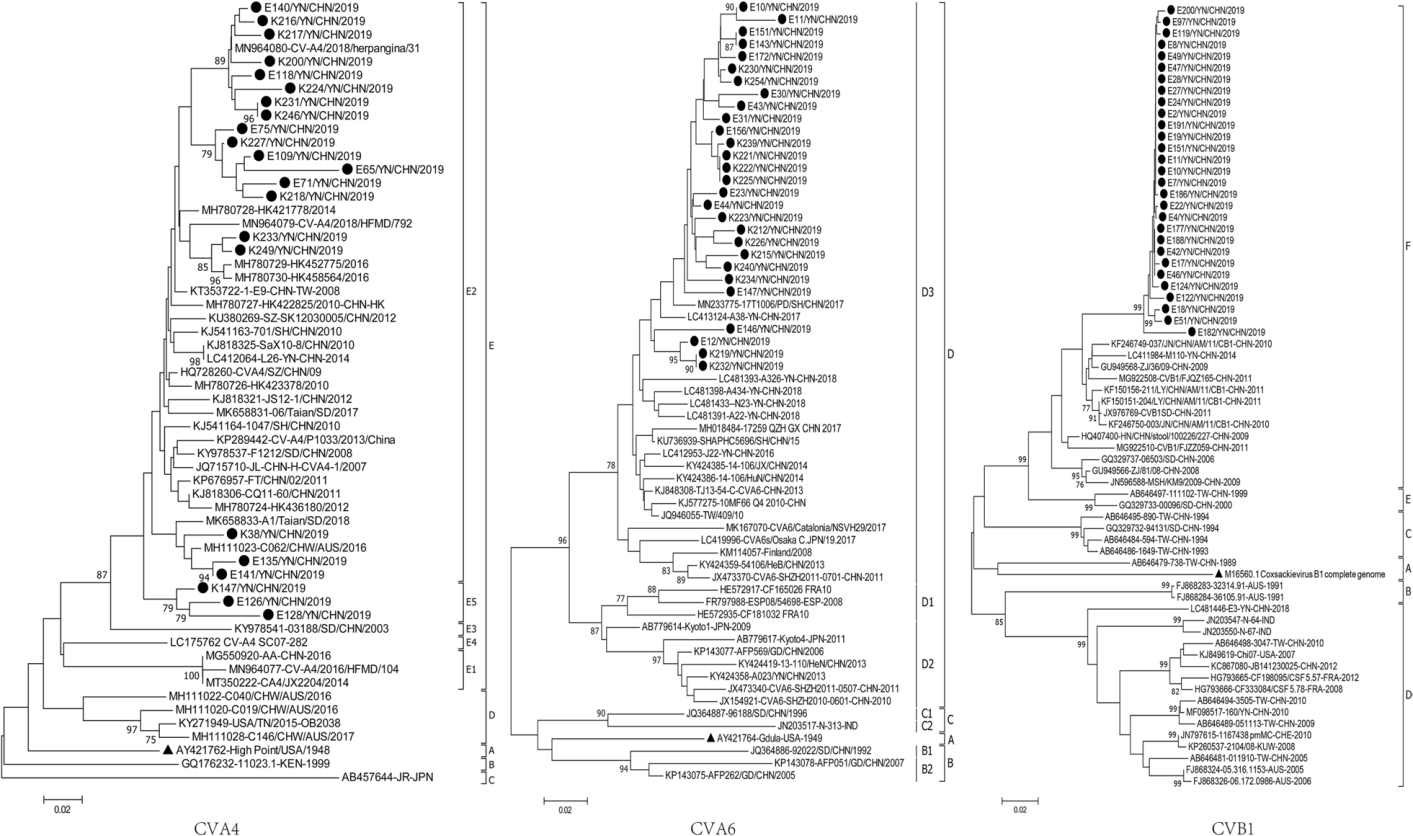
Phylogenetic trees of coxsackievirus B1, coxsackievirus A6, and coxsackievirus A4 based on the partial VP1 sequences by the neighbor-joining algorithm implemented in MEGA (version 7.0) using the Kimura two-parameter substitution model and 1000 bootstrap pseudo-replicates, respectively. Only strong bootstrap values (> 75%) are shown. ● Indicates strains isolated in this investigation; ▲ indicates the prototype strain

The CV-A6 genotypes A, B, C and D were identified and B and C genotypes were further divided into sub-genotypes B1 and 2, and C1 and 2, respectively. Sixty-three sequences, made up of 28 strains from this study and 35 from the GenBank were randomly selected to construct a phylogenetic tree. This showed that CV-A6 isolates in the study belonged to genotype D3 (Fig. 1), which belonged to the predominant strain in mainland China. The nucleotide and amino acid similarities of the partial VP1 gene between the CV-A6 isolates in the study and other Chinese CV-A6 strains were 83.5–95.4% and 94.7–98.9%, respectively.

The CV-B1 strains were divided into A, B, C, D, E and F genotypes, respectively. A phylogenetic tree was constructed from 29 partial VP1 nucleotide sequences that were randomly selected from those of this study and 39 representative strains from GenBank (Fig. 1). All the isolates from this study were of genotype F, which belonged to the main Chinese predominant strains. The nucleotide and amino acid similarities of the partial VP1 gene between the CV-B1 isolates in this study and other Chinese CV-B1 strains within the VP1 gene were 81.3–95.6% and 97.1–100%, respectively.

The phylogenetic analysis of 24 CV-A4 strains in the study and 34 the representative strains (Fig. 1) found that CV-A4 strains were divided into A, B, C, D, and E genotypes. The isolates in the study were part of the E strain, which also belonged to the main Chinese predominant strains. The nucleotide and amino acid similarities of the partial VP1 gene between the CV-A6 isolates in the study and other Chinese CV-A6 strains were 86.0–96.5% and 97.6–98.8%, respectively.

## Discussion

Currently, more than 20 enterovirus serotypes have been associated with HFMD in China and have often co-circulated in outbreaks or in sporadic infection [5, 10]. Most of them are usually detected at low proportions, with EV-A71 decreasing dramatically and CV-A16 still one of the main causative agents. Other EVs such as CV-A6 and CV-A10 are now the main causative agents of HFMD [4, 11], with CV-A4 also frequently detected in HFMD outbreaks [12–14].

This study is the first to report a new HFMD epidemic pattern characterized by co-circulation of three main EV serotypes CV-A6, CV-A4, and CV-B1 in Kunming, with the ratio of co-infection of multiple serotypes significantly higher, which was not consistent with previous reports [4, 5, 10]. In Kunming, EV-A71 and CV-A16 were the main causative agents of HFMD from 2009 to 2015, EV-A71 was the main causative agent in 2009, 2011, 2013, and 2015, but in 2010, 2012 and 2014, CV-A16 was the main causative agent [15]. In 2018, CV-A6, CV-A10 and CV-A16 were the main causative agents [4]. In 2019, CV-A-6, CV-A4, and CV-B1 were the main causative agents and co-circulated in the HFMD outbreak in Kunming. Though EV-A71 had been often associated with severe or fatal cases [4, 12], EV-A71-positive cases have decreased dramatically and led to a significant reduction in the severity and death rate [4, 11]. This reduction in the incidence of EV-A71 may be due to the successful use of the EV-A71 vaccine [16, 17], but the proportion of severe HFMD is still much higher, so even if the number of EV-A71 positive cases is small, monitoring of these cases is still important.

This study also found that CV-A6, CV-A10, CV-A16 and CV-A4 were associated with severe HFMD cases, which was consistent with previous reports [12]. Other EVs such as CV-B1, CV-A9, CV-A5 and E18 were also associated with severe HFMD cases in this study. The ratio of severe HFMD caused by CV-B1, CV-A9 and E-18 was much larger in the group of severe HFMD cases, as was the ratio of multiple serotypes co-infection. In addition, the proportion of co-infection with multiple serotypes is very much higher in the study, which is consistent with previous report [18]. And this pattern of infection might contribute significantly to the clinicopathological progression, thereby influence the incidence and symptoms of the disease [18–21].

Recombination frequently occurs for EVs [22, 23]and several of the current predominant strains have been confirmed as recombinant strains [24–27]. This recombination may occur due to the favorable simultaneous co-infection and replication of the different viruses in the same cell [27]. Although EV-A71 vaccine has been introduced into China, the number of HFMD cases is still very high. The multi-serotypes co-circulate and co-infect simultaneously, and one EV serotype lacks cross immunity protection for patients infected with other serotypes, which may be the reasons that HFMD continues to cause epidemics and outbreaks. This epidemiological feature not only poses a challenge for diagnosis, but also for the prevention and control of HFMD. It is therefore very important to detect more EV serotypes including EV-B for HFMD and the development of multivalent vaccines will be helpful to decrease the morbidity associated with HFMD.

More importantly, the EV-B serotypes are often not the preferred serotype to try to detect in HFMD epidemics. This study found that EV-Bs accounted for 37.70% in the single EV infection HFMD cases, consisting of CV-B1 (21.47%) and CV-A9 (10.99%). In the HFMD outbreaks of Shandong, China in 2009, 17.4% were CV-B strains, 11.8% were CV-B4 and in 2011 [28], CV-B5, CV-A16 and EV-A71 were the three main serotypes [29]. In Korea from 2012 to 2019, a total of 45 EV types were detected from 4399 samples with EV-associated symptoms, with EV-A71 the most detected (15.8%), E-30 (10.0%), CV-B5 (7.8%), CV-A6 (7.6%) and CV-A10 (7.4%). Of these, 46.7% were EV-A and 52.0% were EV-B [30], which indicated EV-Bs were an underestimated pathogen.

Cases due to EV-Bs are mostly asymptomatic or mild, but a few may cause severe issues such as aseptic meningitis, encephalitis, acute myalgia and rhabdomyolysis, pancreatitis and myocarditis, acute flaccid paralysis (AFP) and other serious diseases. Of these, CV-B1 has associated with an increased risk of type 1 diabetes (T1D) and was the predominant EV among neonates in the United States in 2007 [31], with CV-B1 epidemics causing life-threatening neonatal infections [32, 33]. Since the outbreak of HFMD in 2008, CV-B1 was often detected in China [34–36] and CV-B1 accounted for 21.47% in the single EV infection HFMD cases in the study. This indicated the monitoring of CV-B1should be enhanced. Therefore, it is very important to detect more EV serotypes including EV-B for HFMD and the development of multivalent vaccines will be helpful to decrease the morbidity associated with HFMD.

## Conclusion

From 459 HFMD patients in Kunming China in 2019, 18 EV serotypes were identified of which CV-A-6, CV-B1 and CV-A4 were the predominant serotypes. This study presented a comprehensive and detailed investigation regarding the outbreak of HFMD in Kunming based on the pathogenetic and phylogenetic analysis. This will be beneficial for the future prevention and control of HFMD.

## Data Availability

The datasets used and/or analyzed during the current study are available from the corresponding author on reasonable request.

## Abbreviations

EV: Enterovirus
HFMD: Hand foot and mouth disease
EV71: Human enterovirus 71
CV-A16: Coxsackievirus A16
CV-A4: Coxsackievirus A4
CV-A10: Coxsackievirus A10
CV-B5: Coxsackievirus B5
CV-B1: Coxsackievirus B1
CV-A5: Coxsackievirus A5
CV-A9: Coxsackievirus A9
E18: Echovirus 18
E30: Echovirus 30
CV-A6: Coxsackievirus A6
AFP: Acute flaccid paralysis
T1D: Type 1 diabetes
CT: Cycle threshold
SD: Standard deviation
